# Human-Induced Changes in Landscape Configuration Influence Individual Movement Routines: Lessons from a Versatile, Highly Mobile Species

**DOI:** 10.1371/journal.pone.0104974

**Published:** 2014-08-11

**Authors:** Carlos Camacho, Sebastián Palacios, Pedro Sáez, Sonia Sánchez, Jaime Potti

**Affiliations:** 1 Department of Evolutionary Ecology, Estación Biológica de Doñana–CSIC, Seville, Spain; 2 Department of Conservation Biology, Estación Biológica de Doñana–CSIC, Seville, Spain; 3 Department of Environmental Biology and Public Health, University of Huelva, Huelva, Spain; University of Regina, Canada

## Abstract

Landscape conversion by humans may have detrimental effects on animal populations inhabiting managed ecosystems, but human-altered areas may also provide suitable environments for tolerant species. We investigated the spatial ecology of a highly mobile nocturnal avian species–the red-necked nightjar *(Caprimulgus ruficollis)*–in two contrastingly managed areas in Southwestern Spain to provide management recommendations for species having multiple habitat requirements. Based on habitat use by radiotagged nightjars, we created maps of functional heterogeneity in both areas so that the movements of breeding individuals could be modeled using least-cost path analyses. In both the natural and the managed area, nightjars used remnants of native shrublands as nesting sites, while pinewood patches (either newly planted or natural mature) and roads were selected as roosting and foraging habitats, respectively. Although the fraction of functional habitat was held relatively constant (60.9% vs. 74.1% in the natural and the managed area, respectively), landscape configuration changed noticeably. As a result, least-cost routes (summed linear distances) from nest locations to the nearest roost and foraging sites were three times larger in the natural than in the managed area (mean ± SE: 1356±76 m vs. 439±32 m). It seems likely that the increased proximity of functional habitats in the managed area relative to the natural one is underlying the significantly higher abundances of nightjars observed therein, where breeders should travel shorter distances to link together essential resources, thus likely reducing their energy expenditure and mortality risks. Our results suggest that landscape configuration, but not habitat availability, is responsible for the observed differences between the natural and the managed area in the abundance and movements of breeding nightjars, although no effect on body condition was detected. Agricultural landscapes could be moderately managed to preserve small native remnants and to favor the juxtaposition of functional habitats to benefit those farm species relying on patchy resources.

## Introduction

Increasing land-use by humans (e.g. forestry, grazing and agriculture) in recent decades has resulted in the loss, subdivision and reduction in size of large natural areas [1], [2]. Conversion of natural and moderately managed lands into intensively managed landscapes has drastically altered the availability and quality of animal habitats [1], potentially causing population declines [3]–[6]. However, not every species responds equally to land transformation. Landscape management may lead to the appearance of new environments [1] that may enhance landscape heterogeneity and thus provide suitable habitats for species tolerant to anthropogenic alterations [7]–[11]. In this context, species’ tolerance to anthropogenic changes emerges as a key feature influencing their persistence in agricultural systems [12]–[14].

In human-dominated areas, landscape management affects landscape heterogeneity through changes in landscape composition and configuration (i.e. respectively, the number and proportion of different cover types and their spatial arrangement) [14]. Landscape heterogeneity can vary widely, as certain cover types may be selectively retained while others are lost according to the criteria of individual land owners, and habitat patches can be either interspersed or occur in extensive blocks, contiguously or separated by unsuitable habitat [15]. Even when the fraction of usable habitat for fauna is held constant, modification of the size and arrangement of habitat patches can strongly influence the configurational heterogeneity of a landscape. This would determine landscape complementation [16], defined as the process by which proximity of landscape elements enables individuals to link together critical habitat types (i.e. containing essential resources) through movement [17]. Many species move across multiple habitats on a daily basis [18] and the costs of these movements (e.g. increased energy expenditure and mortality risk) increase with the time spent moving [19]. As a consequence, human modification of landscape structure can influence a variety of ecological responses, including population density [20] and persistence [13], as well as animal movements [21]. In this context, recent studies have shown that highly mobile organisms can cope to some extent with −or even benefit from− moderate landscape modifications (e.g. butterflies [22], felids [23], bats [24] and raptors [25]). For example, open-habitat birds may not always respond to habitat loss or conversion by showing rapid declines, but instead each species will respond differently, depending on the availability and arrangement of spatial resources at the landscape scale [16], [26]. It is well known that shifts from large, highly productive uniform fields to natural or extensively managed lands can increase habitat connectivity and enhance biodiversity, and the importance of habitat mosaics for animal species is widely recognized [20], [27], [28]. However, specific patterns of landscape management aimed at promoting biodiversity without reducing agricultural production remain largely unexplored.

The Red-necked Nightjar *(Caprimulgus ruficollis*; henceforth nightjar*)* is a long-distance migrant that inhabits dry warm regions in northern Africa and southwestern Europe [29]. Nightjars are associated with open natural and agricultural areas, but use different complementary habitats to fulfil their life-history requirements (see results). From dusk to dawn, nightjars use bare open areas for hunting flying insects because such habitats facilitate prey and predator detection [30] and may provide some thermal benefits [31], [32]. However, nightjars have different habitat requirements for nesting and roosting, and adults typically commute from nesting areas in open shrublands or cropland [33] to daylight roosts in shaded woodlands. Nesting, roosting and foraging habitats are quite different and therefore usually located some distance apart, producing a scenario where the potential costs of commuting may be readily detectable [34]. Nightjars’ use of distinctly different habitat types both within managed and unaltered environments therefore provides a good opportunity to investigate the effects of human-induced changes to habitats on the extent and nature of bird movement [35].

We used information from radio-tagged nightjars to assess the spatial responses to landscape transformation by birds breeding in two highly contrasting environments (man-made patchy *vs.* natural clumped distribution) as a result of unequal land protection policies (managed private property *vs.* highly protected area). Specifically, we hypothesized: (1) that structural differences following landscape transformation would force nightjars in the managed area to alter habitat selection patterns relative to those inhabiting the unaltered area; (2) that changes in the availability and the spatial arrangement of functional habitats in the managed area would influence the length of daily movements by nightjars to meet habitat requirements; and (3) that increased daily travel distances would negatively affect the body condition of breeding individuals. To test these predictions, we analysed nightjars’ selection of nesting, foraging and roosting habitats in both areas and quantified the extent to which habitat availability and configuration affect landscape use and daily movements, as well as the body condition of breeding individuals. Finally, we aimed to identify the implications of landscape changes for the conservation of species with multiple habitat requirements to provide management guidelines.

## Methods

### Ethics Statement

This research was licensed by the Andalusian Authority for Wildlife Protection (permit numbers: 4358/1064/MDCG and 762/MDCG). This research required that birds were subjected to minimal disturbance (collection of biometric data and radiotransmitters’ attachment) and birds were not released until we assessed their welfare. This study did not involve protected, threatened or endangered species and was carried out according to national and international guidelines. The Ethics Committee on Animal Experimentation from Doñana Biological Station−CSIC approved the animal handling procedures (ref. 1/1988_2).

### Study system

The study was conducted from March to November 2011 and 2012 in Doñana National Park and nearby areas (southwestern Spain). Based on preliminary observations from August to November 2009 and 2010, we selected two close (10 km) but highly contrasting plots in terms of disturbance and protection regime to assess patterns of habitat use by nightjars: the Doñana Biological Reserve (37°0′N, 6°30′W), a natural area within the protected core of the Doñana National Park, and a managed property (37°8′N, 6°34′W), neighboring the northwestern border of the National Park. The natural area is characterized by heterogeneous plant communities that include large expanses of Mediterranean shrublands dominated by *Halimium halimifolium, Ulex spp.* and *Erica spp*. with scattered patches of *Juniperus phoenicea* and *Pinus pinea*. The managed area is mostly characterized by regularly-shaped blocks of habitat that include small, undisturbed remnants of Mediterranean shrublands, cattle-grazed pastures, extensively managed pinewood patches, and intensively managed plantations of orange trees. In contrast to the natural area, where human access and activities are highly restricted, the managed site has no protection status and resource exploitation (i.e. agriculture, forest tree crops, cattle raising and hunting) are common activities.

### General field procedures

We conducted weekly transect counts of road–sitting nightjars by driving a vehicle at a constant speed of 30 km/h, beginning 1−2 h after dusk (see [32] for details). Nightjars were captured along roads using a LED torch and a hand-held net [36]. Capture–mark–recapture (CMR) models show that the fraction of the whole population sampled during the systematic vehicle transects along roads is similarly high in both study areas (73% and 78% of all the individuals in the managed and the natural area, respectively, *χ^2^_1_* = 0.20, *P* = 0.68; authors’ unpubl. data). Therefore, we are confident that our estimates were not biased by the sampling procedure. All individuals were uniquely marked with numbered metal rings, sexed according to the pale spots on flight feathers, and aged as either yearling or older following [37]. Birds were weighed (±0.1 g) and we measured keel length (±0.01 mm), a reliable predictor of skeletal size [38]. We used body mass and keel length to assess body condition (i.e. body mass, controlling for size) and body size, respectively. Palpation of the abdomen (scored as full, ¾, ½, ¼ or empty) [30] provided an estimate of the amount of food contained, and subcutaneous fat stores were visually ranked from 0 (no visible fat) to 4 (belly covered with fat) following a standardized scale modified from [39].

### Radiotracking

During the 2011 and 2012 breeding seasons (i.e. May-August) [40], we fitted thirteen adult nightjars with conventional radio transmitters (PIP3– Biotrack Ltd UK; <2% of body mass) glued onto the central tail feathers (Table 1). We used data on timing of breeding [40] to ascertain the tagging dates and only tagged gravid females or adult individuals of either sex showing an active brood patch to ensure that their habitat requirements were comparable [41], [42].

**Table 1:** Tracking parameters for the 13 radiotagged individuals.

Individual	Sex	Area	Year	Tracking effort (No. sessions)	Tracking period (days)	No. fixes
270	Female	Managed	2011	23	64	50
270^a^ *	Male	Managed	2012	4	6	11
538	Female	Managed	2012	21	73	64
705	Female	Managed	2012	18	55	60
798	Male	Managed	2012	16	49	55
894	Male	Managed	2012	12	50	45
950^b^ *	Female	Managed	2011	3	14	3
621	Male	Managed	2011	29	53	59
342	Female	Natural	2011	25	65	47
680^c^	Male	Natural	2012	13	30	46
734	Female	Natural	2012	18	43	54
933	Female	Natural	2011	13	57	45
981	Male	Natural	2012	18	43	53

Individual tracking code, sex, area, year, tracking effort (number of sessions), tracking period (days) and total number of fixes are shown.

a Transmitter (re-utilized) failed after ≥8 days but was still on the bird when recaptured 74 days later.

b No signal was received in the following 50 days in a 1000 m radius from the capture location.

c No nest was found, hence being excluded from further analyses.

*Excluded from further analyses as the number of fixes was insufficient to represent a complete home range.

We tracked radiotagged individuals every other night for 1–2 h sessions using a 3-element Yagi-antenna connected either to an ICOM IC-R20 (http://www.icom.co.jp) or a SIKA (Biotrack Ltd, UK) portable receiver. Tracking sessions did not begin until at least 24 h after tagging and were scheduled to collect data throughout the complete night period for each individual. Individuals were tracked beyond the nesting period, until feeding associations between adults and young finished (range 43–73 days; see [40]), even when the minimum number of fixes required for an accurate estimation of home range size (24±9 (SD) fixes) had been attained. Intermittently, nightjars were followed continuously from adjacent habitat types with two portable receivers being simultaneously used for 4–8 h periods to assess their movement routes through the landscape matrix. The effective range of transmitter signals was 400−600 m and the mean accuracy of fixes was *x = *35±17 (SD) m (range = 11–52), calculated after a biangulation of individuals. Foraging birds sometimes remained in the same location up to 30 min, so we only recorded new fixes after at least 1 h passed or birds had moved beyond the minimum accuracy of fixes (52 m) to minimize sample clustering [43]. Fixes were determined either by biangulation with the software LOAS (ESS, LLC.) or through direct sighting of individuals on roads. We recorded *in situ* the location of birds encountered on roads with a Garmin GPS 60 (2–4 m accuracy) at >1 h intervals to avoid bias due to relocating birds disturbed by the observer [44]. We recorded the location of nests and roosts during diurnal tracking sessions [45].

### Landscape mapping and habitat selection

To assess the functional landscape heterogeneity for nightjars, we used the procedure described in [14]. First, we created a map of different cover types (i.e. structural heterogeneity) present in the managed and the natural area (2059 and 4857 has, respectively) and determined the spatial resources required by nightjars (see below). Next, we grouped together sets of patches containing the same resource type to create a landscape map of functional habitats from which measures of compositional and configurational heterogeneity can be extracted.

To map structural landscape heterogeneity, we superimposed a high-resolution (0.5 m) vegetation map (www.juntadeandalucia.es/medioambiente/rediam) over an ortho-photograph at the same resolution (www.juntadeandalucia.es/institutodeestadisticaycartografia) using the ArcGIS 10 software (ESRI 2010). Data from the ortho-photograph were ground-truthed to refine with subsequent digitalization. A total of 205 different land cover types were grouped into 10 cover typologies according to vegetation composition (either natural or managed) and human activity levels (Table S1).

To map functional landscape heterogeneity, we grouped cover typologies according to the habitat selection patterns of nightjars. For this purpose, we first calculated individual home range areas (see ‘statistical analyses') and superimposed them over the structural map using the ArcGIS 10 software. Then, we compared the observed locations of individuals having a particular behavior (nesting, roosting or foraging) to habitat availability (surface) within individual home ranges [46]. The map of functionally different cover types consisted of four main habitat categories: (1) breeding habitat, considered as that covered by open shrublands; (2) roosting habitat, including pinewood forests (both natural mature and newly planted patches); (3) foraging habitat, including paved and gravel roads (both areas) as the main foraging sites, and sandy paths (only in the natural area) and orange tree plantations (only in the managed area) as secondarily used foraging sites; (4) non-usable habitat, mapped as a single cover type that includes all the environments in which no directionality of selection was detected (Table S1). At a final step, we used this map to calculate the fraction of usable habitat within each study plot and to assess interspersion of critical habitat types.

### Effect of landscape configuration on nightjar movements

To assess the extent to which landscape configuration influences the movement needs of nightjars, we quantified interspersion of functional habitats in both study sites using Morańs index [47], [48] as calculated in ArcGIS 10, and then modelled the daily movement needs of nightjars (i.e. summed distances from simulated nests to the nearest roosting and foraging habitats) through least-cost analyses [49], assuming that (1) breeding individuals flew at minimum on a daily basis, the straight route from nests to the nearest roost and foraging site [18]; and (2) that movement distances in patchy landscapes typically match the linear distances between habitat patches to minimize movement costs [21]. Nest locations (hereafter, nests) were simulated with the tool ‘*create random points*’ in ArcGIS 10 by randomly spreading nests at a minimum distance of 50 m [33] among the patches of breeding habitat that we had previously defined in analyses of habitat selection in both study areas [50]. Numbers of nests simulated in both plots (*n* = 60) matched the estimated breeding density (i.e. maximum number of different females with a brood patch recorded throughout a complete breeding season) in the less densely populated plot (i.e. the natural area; authors’ unpubl. data further validated with CMR models).

### Statistical analyses

All statistical analyses were done using R 2.14.0 (http://www.r-project.org). We calculated individual home range areas using bivariate normal kernel functions [51], using the function ‘*kernelUD*’ in the package ‘adehabitatHR’ [52]. In contrast to the MCP method, which simply calculates the area bounded by the outermost locations, kernel analyses provide a probability density function estimating the likelihood of an animal being present within a two-dimensional plane, so that potential bias due to unusual, large movements can be avoided [45]. The smoothing parameter *(h)* controlling the bandwidth of the kernel function was set at 200 after successive exploratory trials [52]–[54]. To analyze patterns of habitat selection we compared the observed locations in each cover type to those expected under random habitat choice using Chi-square tests and then calculated the 95% confidence intervals for the proportion of locations within respective land cover types following [55]. A land cover type was considered to be selected (or avoided) by nightjars when the observed proportion of locations was below (or above) the confidence limits [44].

**Figure 1:**
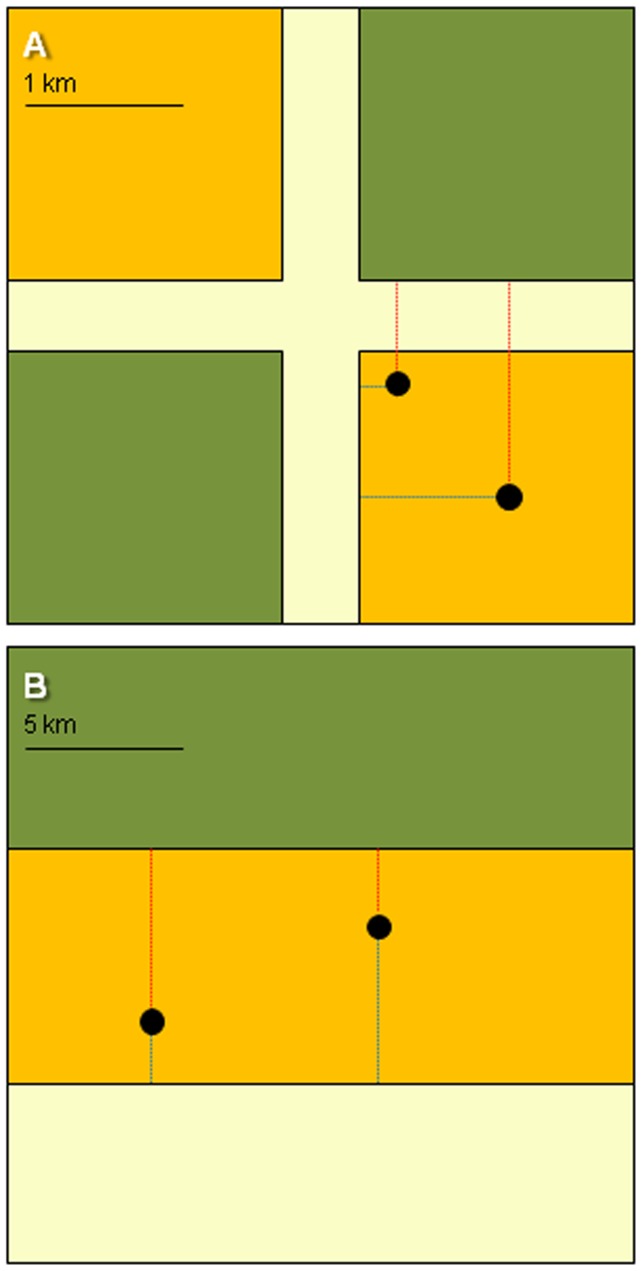
Schematic view of the predicted least-cost movements of breeding individuals in contrastingly managed landscapes. Nesting (orange), roosting (green) and foraging (yellow) habitats are shown. **1a.** Breeding habitat patches are arranged regularly as small-sized blocks and a close juxtaposition of functional habitats exists. As a result, distances from nests (black dots) to foraging sites (blue lines) are expected to increase with nest-to-roost distances (red lines). **1b.** Functional habitats are clumped as comparatively large-sized blocks. As a result, distances from nests to foraging sites are expected to increase as distances between nests to roosts decrease. Note that, despite the fraction of functional habitat is held constant in both areas, mean distances between nests and the other two habitat types are longer in 2b than in 2a.

**Figure 2:**
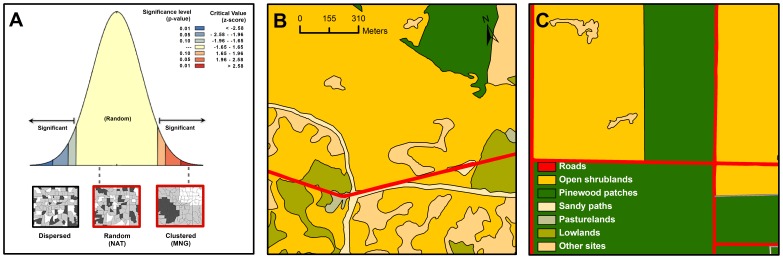
Spatial configuration of functional habitats for nightjars in the natural (clumped) and the managed area (random). 2a. Results from the Morańs *I* index. Sections of the natural (**2b**) and the managed area (**2c**) maps illustrating these differences in landscape configuration are also shown.

A visual inspection of the land cover maps suggested that the linear nest-to-roost and nest-to-foraging site distances correlate differently with habitat clumping (Fig. 1 and 2). To test this, we calculated bootstrap correlations (nest-to-roost and nest-to-foraging site distances; 10,000 replacements) and then tested for differences between correlations using the function *paired.r* in the package ‘psych’ [56]. To quantify differences in the daily movement needs of nightjars between the two plots, we used a General Linear Model (GLM; Poisson errors, log link function), including the summed Euclidean distances between functionally different habitats (i.e. nest-to-roost+nest-to-foraging site distances) as the response variable and the study area as a fixed effect.

To assess whether body condition is affected by landscape configuration, we fitted a General Linear Mixed Model (GLMM; normal errors and identity link function) including body mass as the response variable and keel length as a covariate to control for body-size-dependent variation in body mass. Fat stores and stomach volume were included as covariates in the model and sex was entered as a fixed effect. Year and individual identity were included as random effects to account for repeated measures of the same individuals and annual heterogeneity. Gravid females (<5%) and potential migrants, considered as those recorded exclusively beyond 20 August [40] were omitted from the analysis. The GLMM was fitted using the function *lmer* in the package ‘lme4’ [57]. To achieve *P*-values, we fitted the model using Maximum Likelihood [58]. *P*-values for the individual effects were based on Markov Chain Monte Carlo (MCMC) sampling (10,000 iterations) and derived using the function *pvals.fnc* in the package ‘languageR’ [59].

To test for differences in bird abundance between both study areas, nightjar numbers were standardized to birds/km and compared using the Wilcoxon signed-rank test *(T)*. Weeks in which values of nightjar abundance were zero in both plots were omitted from the analysis. To increase the data set of bird measurements and nocturnal counts, data from 2009−2010 were also included in this analysis. All tests were two-tailed.

## Results

### Habitat selection patterns and functional heterogeneity

Altogether, we obtained 592 locations from 13 different nightjars during 53 sessions of radiotracking activity (Table 1). However, usable data were available for only 10 of the 13 birds due to insufficient numbers of fixes for two birds and the failure to locate the nest of one additional bird (possibly loss due to predation on eggs or chicks shortly after tagging; Table 1).

Nightjar activity was dependent on land cover types (Table 2). In both the natural and the managed area, all focal individuals placed their nests within open shrublands (*n* = 10 nests), while roosting nightjars (except for females incubating or brooding recently-hatched chicks) were only recorded within pinewood patches during daylight (*n* = 142 daytime fixes). Cover-type dependence also extended to the foraging behavior, and gravel and paved roads were strongly selected as the main foraging habitat, except for one female (#734) for which road use was only anecdotally recorded (Table 2 and Table S1). Despite the remarkably small surface (<1%) covered by roads, 31% of foraging locations in both areas were on road.

**Table 2:** Patterns of habitat selection by red-necked nightjars.

Individual	Area	Selected nestinghabitat	Selected roostinghabitat	Selected foraginghabitat	Avoided foraginghabitat	Model *P-* *value*
270	Managed	Open shrublands	Pinewood patches	Roads	Pinewood patches	*0.056*
538	Managed	Open shrublands	Pinewood patches	Roads	Pinewood patches, open shrublands	*<0.001*
621	Managed	Open shrublands	Pinewood patches	Roads	Open shrublands	*0.026*
705	Managed	Open shrublands	Pinewood patches	Roads, orange tree crops	Pinewood patches	*0.002*
798	Managed	Open shrublands	Pinewood patches	Roads	Pinewood patches, open shrublands	*0.001*
894	Managed	Open shrublands	Pinewood patches	Orange tree crops	None	*0.526*
342	Natural	Open shrublands	Pinewood patches	Roads	Open shrublands	*0.012*
734	Natural	Open shrublands	Pinewood patches	Roads, sandy paths	None	*0.115*
933	Natural	Open shrublands	Pinewood patches	Roads	Open shrublands, other sites	*<0.001*
981	Natural	Open shrublands	Pinewood patches	Roads	None	*0.022*

Nesting, roosting and foraging habitats of radio-tagged individuals breeding in the managed and the natural area are shown. Directionality of selection is summarized from Table S1.

Native open shrublands were the main land cover type in the natural area (47.5% of total surface), whereas the managed area was mainly covered by pinewood plantations (54.3% *vs.* 14% of native remnants) as a result of landscape conversion by humans. Nonetheless, the fraction of usable habitat for nightjars at the landscape scale in the natural area (60.9%) was not significantly different from that in the managed site (74.1%; *χ* ^2^_1_ = 3.2, *P = *0.07). In contrast, landscape configuration of both plots was markedly different: functional habitats were clustered in the natural area (Morańs *I = *0.09, *Z*-score = 3.22, *P*<0.01) and randomly distributed in the managed area (Morańs *I = *0.10, *Z*-score = 0.61, *P* = 0.54; Fig. 2a).

### Effect of landscape configuration on nightjar movements and abundance

Except for females incubating or brooding recently-hatched chicks, which remained in the nest during daylight, breeding nightjars moved daily from nests to (usually the nearest) roosts and foraging sites. The simultaneous use of two different receivers located in adjacent habitat types allowed us to determine that, at least for continuously monitored individuals, actual movements between functionally different habitats were typically direct flights.

**Figure 3:**
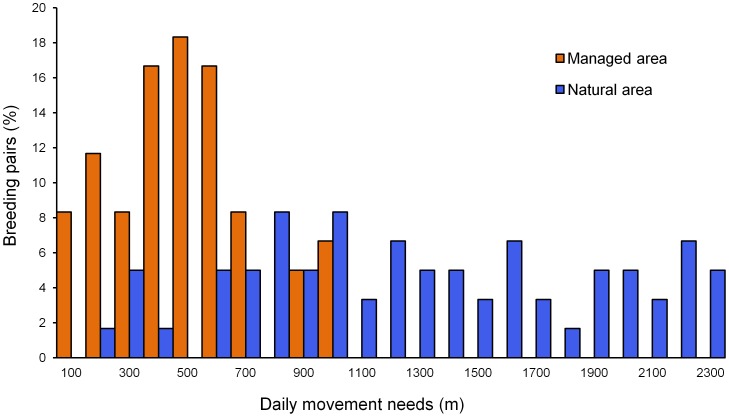
Distribution histograms for the modeled movements needs of nightjars breeding in both study areas. Movement needs of a breeding pair reflect the summed distances from each nest to the nearest roosting and foraging habitats.

Estimated least-cost movements (mean ± SE) from nests to the nearest roost and foraging sites in the natural area (478±41 m and 878±62 m, respectively) were three times longer than those in the managed area (157±18 m and 282±21 m, respectively; GLMs with Poisson errors testing separately for differences between both study sites in nest-to-roost and nest-to-foraging site distances; both *P<*0.0001). Our analyses of the observed least-cost movements by the focal individuals yielded similar results (data not shown; permutation *t*-tests, both *P*<0.0001), suggesting that habitat clumping in the natural area leads to breeding nightjars performing longer trips to fulfil daily habitat requirements than in the managed area (Fig. 3). As expected after visual inspection of the land cover maps (Fig. 1), distances from nests to the nearest foraging sites were positively correlated with distances to the nearest roost in the managed area (*r_bs_* = 0.38; 95% CI: 0.17, 0.58), whereas no such trend was observed in the natural area (*r_bs_* = 0.06; 95% CI: −0.17, 0.29; test of difference between two independent correlations: *Z* = 1.88, *P* = 0.06).

**Figure 4:**
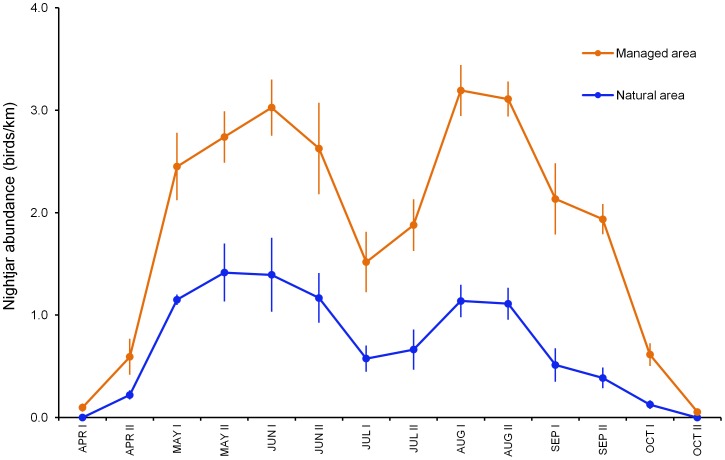
Seasonal variation (mean ± SE) in the abundance of nightjars (birds/km). Estimated values for the managed and the natural area between 2009 and 2012 are shown by half month (I, first half; II, second half).

Nightjar abundance was consistently higher in the managed than in the natural area (Wilcoxon signed-rank test with continuity correction: *T = *2475, *P*<0.0001, *n* = 74 paired counts; Fig. 4), where bird occurrence also reached the highest time-point values (2.9 *vs.* 2.2 birds/km in the managed and the natural area, respectively). However, the body condition of breeders did not differ between areas (*n = *328 measurements of 193 individuals; estimate ± SE body mass = 0.73±1.08, *P*_MCMC_ = 0.75, after controlling for the significant effect of body size, sex, fat and stomach volume, all *P*_MCMC_ <0.01).

## Discussion

Our results indicate that Red-necked Nightjars have multiple habitat requirements during the breeding season irrespective of the degree of land management. In both the natural and the managed area, radiotagged individuals used open shrublands as nesting sites and pinewood patches as daytime roosts, while roads were selected as the main foraging habitat. Breeding individuals moved on a daily basis from nests to foraging sites and daylight roosts but, as a consequence of habitat clumping, nightjars in the natural area travelled longer distances than those in the managed area.

In the Doñana region, changing land-use policies during the last 30 years have led to intensification of land use, and differences in structural landscape heterogeneity now exist between natural ecosystems and the managed, non-protected areas [60], [61]). However, these differences have not translated into marked dissimilarities in the functional landscape heterogeneity for nightjars, primarily because of the species’ ability to exploit both newly created environments and former natural habitats to obtain essential resources. From this, we predict that nightjars should be more resilient to changes in the landscape than other avian species with more rigid habitat requirements [13], and the use of certain man-made structures to complete their life cycles could enable them, and likely other species as well, to eventually benefit from changes in land use [24], [25], [62].

Our occasional recording of complete flights between adjacent habitat types indicates that nightjar daily trips to obtain necessary resources appear to be straight, although exact movement routes could not be observed in most cases. Movement paths between different habitat types tend to be direct when animals move through low resource cover types to minimize the time spent there [21]. Accordingly, and under the assumption that breeding individuals use straight movement paths between their nearest roost and foraging sites to nests (i.e. least-cost movement), daily movement needs of nightjars appear to be larger in the natural area than in the managed site. Extensive blocks of unsuitable habitat in the natural area might force most breeders to make comparatively longer trips (0.5−2 km) between their nests and either their nearest roost or foraging sites. In contrast, the closer juxtaposition of functional habitats for nightjars in the managed property enabled individuals to fly shorter distances (<0.5 km) to access critical habitat types. Accordingly, differences in landscape configuration (i.e. unequal size and interspersion of habitat patches), but not in landscape composition (i.e. similar fraction of functional habitats), appears to be underlying the observed differences in the movement ecology of nightjars.

Red-necked nightjars are highly mobile and rather tolerant to anthropogenic alterations and hence able to exploit patchily-distributed resources from contrasting environments. The increased proximity of foraging and breeding sites in the managed area apparently allows these birds to link together functionally different habitats, likely reducing energy expenditure and mortality risk [19], [21] leading to a higher density in the managed property [63], [64]. The differences we have found in nightjar numbers between the managed property and the undisturbed natural area as a result of human-caused changes in landscape configuration agree with previous studies showing the positive influence of functional connectivity on animal abundance [20], [62], [65] and support the emerging view that some species may respond differently to land-use intensification and somehow benefit from moderate habitat disturbance [9], [24], [25]. The high nightjar abundance in the managed area might actually represent a positive response to configurational landscape heterogeneity through increased landscape complementation [14], [16]. However, caution is required when interpreting these results because (1) our sample size is actually limited to one of each type of study area, thus limiting our power of inference, (2) land cover use within the landscape matrix is based on least-cost model predictions of movement and (3) functional habitats are defined from a restricted (but we think representative; see Table S1) sample of individuals. Following [17], we assume that a ‘chessboard’ landscape should increase landscape complementation by facilitating movement among equal amounts of different, non-substitutable habitats. However, although results from combined habitat selection analyses and movement simulations are suggestive, the indirect assessment of daily bird trips does not rule out other possibilities. For example, nightjars might not perform linear trips [49] or either could use particular foraging or roosting sites, but not the nearest ones. Moreover, the distance and frequency of foraging trips might be uneven between areas [11]. Consequently, the strongest supporting evidence should be gathered by quantifying complete individual trips to obtain data on actual movement paths and movement risks in the two landscape contexts [21]. In our study this was not feasible due to a mass restriction on the data logger device.

Contrary to our expectations, there was no statistically detectable effect of landscape configuration on the body condition of breeding individuals. A possible explanation is that peaks of aerial insect abundance recorded in the natural (but not in the managed) area during the main breeding season of nightjars (authors’ unpublished data) might counteract the energy costs of increased movement needs therein and thus be masking the potential effect that habitat clumping could have on the body condition of breeding nightjars. A second, non-exclusive hypothesis is that, whereas a close juxtaposition of different usable habitats is probably crucial to enable non-flying and less mobile animals to access essential resources, the presence of large blocks of unsuitable habitat may not have an obvious impact on highly mobile species [18]. If the latter hypothesis is true, then the apparently high mobility and plasticity in habitat use of nightjars might allow them to cope with or even benefit from human-induced changes in landscape composition and configuration while keeping body condition unaltered, thus increasing their chance of persistence in moderately disturbed environments [2], [66].

### Implications for conservation and management guidelines

Conservation of unaltered habitats within natural reserves is commonly considered of major importance for enhancing species diversity in fragmented landscapes but, as shown here and recently by e.g. [24], [25], [62], human-dominated areas may also have great conservation value for at least some bird communities. Despite the risks of using least-cost path analysis for land management decisions lacking detailed data on actual movement paths in the landscape [21], our results support the view that moderately disturbed and fragmented habitats might become valuable sustainers for the red-necked nightjar as well as for other species tolerant to human alterations [25], [62]. Consistent with recent investigations on other related (e.g. raptors) [11] and unrelated (e.g. bats) [24] highly mobile species, our results suggest that (1) high plasticity in habitat choice might increase the chances of species persistence in human-dominated areas [2], [66]; and (2) agricultural landscapes maintaining functional habitats in a mosaic-like arrangement would facilitate bird access to high-quality spatial resources [62]. Therefore, we conclude that (1) ideally, any effort to establish wide-range systems of functional nature reserves should fully engage the private property sector in order to include key landscapes outside protected areas [67]; (2) some undisturbed remnants of native vegetation should be left to favor open–habitat bird species [16], [26]; (3) the juxtaposition of native remnants and human-made structures devoid of vegetation (e.g. trails or roads), coupled with moderately managed forests or pastures would benefit those species relying on multiple, patchy resources in agricultural landscapes.

## Supporting Information

Table S1 **Patterns of foraging habitat selection by radio-tagged red-necked nightjars breeding in the managed (MNG) and the natural (NAT) area.**
(DOCX)
