# Supplementary material for: Human-Induced Changes in Landscape Configuration Influence Individual Movement Routines: Lessons from a Versatile, Highly Mobile Species
Source: PLoS One. 2014 Aug 11;9(8):e104974. doi: 10.1371/journal.pone.0104974 (PMC4128739; doi:10.1371/journal.pone.0104974)
Supplement: Table S1 — Patterns of foraging habitat selection by radio-tagged red-necked nightjars breeding in the managed (MNG) and the natural (NAT) area. (DOCX) [file pone.0104974.s001.docx]

Supporting Information

**Table S1.** **Patterns of foraging habitat selection by radio-tagged red-necked nightjars breeding in the managed (MNG) and the natural (NAT) area.**

| **Bird** | **Area** | **Land cover type** | **Proportion of**  **surface covered** | **Observed**  **locations** | **Expected**  **locations** | ***χ^2^*** | ***P*** | **Obs/Total** | **Lower**  **95% CI** | **Upper**  **95% CI** | **Direction**  **of selection** |
| --- | --- | --- | --- | --- | --- | --- | --- | --- | --- | --- | --- |
| 270 | MNG | Pinewood patches | 0.586 | 7 | 14.660 | 3.61 | 0.057 | 0.280 | 0.141 | 0.478 | Avoided |
|  |  | Human facilities | 0.001 | 0 | 0.030 | 0 | 1 | 0 | 0 | 0.158 | None |
|  |  | Open shrublands | 0.157 | 3 | 3.910 | 0 | 1 | 0.120 | 0.033 | 0.308 | None |
|  |  | Orange tree crops | 0 | 0 | 0.010 | 0 | 1 | 0 | 0 | 0.158 | None |
|  |  | Other sites | 0.019 | 0 | 0.470 | 0 | 1 | 0 | 0 | 0.158 | None |
|  |  | Pasturelands | 0.224 | 4 | 5.590 | 0.04 | 0.832 | 0.160 | 0.058 | 0.353 | None |
|  |  | Roads | 0.012 | 11 | 0.300 | 10.76 | 0.001 | 0.440 | 0.266 | 0.629 | Selected |
|  |  | Sandy paths | 0.001 | 0 | 0.020 | 0 | 1 | 0 | 0 | 0.158 | None |
|  |  | *TOTAL* | *1* | *25* | *25* | *13.75* | *0.056* | *1* |  |  |  |
| 538 | MNG | Pinewood patches | 0.405 | 8 | 19.060 | 5.25 | 0.022 | 0.170 | 0.086 | 0.304 | Avoided |
|  |  | Human facilities | 0.004 | 0 | 0.170 | 0 | 1 | 0 | 0 | 0.090 | None |
|  |  | Open shrublands | 0.128 | 1 | 6.020 | 2.49 | 0.115 | 0.021 | 0 | 0.121 | Avoided |
|  |  | Orange tree crops | 0.180 | 12 | 8.440 | 0.41 | 0.522 | 0.255 | 0.151 | 0.396 | None |
|  |  | Other sites | 0.015 | 0 | 0.720 | 0 | 1 | 0 | 0 | 0.090 | None |
|  |  | Pasturelands | 0.256 | 8 | 12.030 | 0.58 | 0.445 | 0.170 | 0.086 | 0.304 | None |
|  |  | Roads | 0.010 | 17 | 0.490 | 16.90 | <0.001 | 0.362 | 0.239 | 0.505 | Selected |
|  |  | Sandy paths | 0.002 | 1 | 0.070 | 0 | 1 | 0.021 | 0 | 0.121 | None |
|  |  | *TOTAL* | *1* | *47* | *47* | *26.82* | *<0.001* | *1* |  |  |  |
| 621 | MNG | Pinewood patches | 0.650 | 23 | 22.740 | 0 | 1 | 0.657 | 0.491 | 0.792 | None |
|  |  | Human facilities | 0.002 | 0 | 0.070 | 0 | 1 | 0 | 0 | 0.118 | None |
|  |  | Open shrublands | 0.136 | 0 | 4.770 | 3.20 | 0.074 | 0 | 0 | 0.118 | Avoided |
|  |  | Other sites | 0.029 | 0 | 1.000 | 0 | 1 | 0 | 0 | 0.118 | None |
|  |  | Pasturelands | 0.168 | 3 | 5.860 | 0.45 | 0.504 | 0.086 | 0.022 | 0.231 | None |
|  |  | Roads | 0.015 | 9 | 0.520 | 6.80 | 0.009 | 0.257 | 0.140 | 0.423 | Selected |
|  |  | Sandy paths | 0.001 | 0 | 0.020 | 0 | 1 | 0 | 0 | 0.118 | None |
|  |  | *TOTAL* | *1* | *35* | *35* | *14.34* | *0.026* | *1* |  |  |  |
| 705 | MNG | Pinewood patches | 0.322 | 1 | 12.220 | 9.56 | 0.002 | 0.026 | 0 | 0.147 | Avoided |
|  |  | Human facilities | 0.004 | 0 | 0.150 | 0 | 1 | 0 | 0 | 0.109 | None |
|  |  | Open shrublands | 0.252 | 9 | 9.560 | 0 | 1 | 0.237 | 0.128 | 0.394 | None |
|  |  | Orange tree crops | 0.275 | 25 | 10.440 | 9.72 | 0.002 | 0.658 | 0.498 | 0.789 | Selected |
|  |  | Other sites | 0.031 | 0 | 1.200 | 9.54 | 0.002 | 0 | 0 | 0.109 | None |
|  |  | Pasturelands | 0.107 | 0 | 4.060 | 2.44 | 0.119 | 0 | 0 | 0.109 | None |
|  |  | Roads | 0.008 | 3 | 0.300 | 0.92 | 0.339 | 0.079 | 0.020 | 0.215 | Selected |
|  |  | Sandy paths | 0.002 | 0 | 0.070 | 0 | 1 | 0 | 0 | 0.109 | None |
|  |  | *TOTAL* | *1* | *38* | *38* | *23.21* | *0.002* | *1* |  |  |  |
| 798 | MNG | Pinewood patches | 0.493 | 11 | 17.730 | 1.90 | 0.168 | 0.306 | 0.179 | 0.470 | Avoided |
|  |  | Human facilities | 0.003 | 0 | 0.110 | 0 | 1 | 0 | 0 | 0.115 | None |
|  |  | Open shrublands | 0.224 | 2 | 8.060 | 2.96 | 0.085 | 0.056 | 0.006 | 0.191 | Avoided |
|  |  | Orange tree crops | 0.153 | 4 | 5.490 | 0.03 | 0.865 | 0.111 | 0.038 | 0.259 | None |
|  |  | Other sites | 0.043 | 0 | 1.530 | 0.19 | 0.665 | 0 | 0 | 0.115 | None |
|  |  | Pasturelands | 0.076 | 1 | 2.740 | 0.15 | 0.694 | 0.028 | 0 | 0.154 | None |
|  |  | Roads | 0.008 | 18 | 0.270 | 20.53 | <0.001 | 0.500 | 0.345 | 0.655 | Selected |
|  |  | Sandy paths | 0.002 | 0 | 0.060 | 0 | 1 | 0 | 0 | 0.115 | None |
|  |  | *TOTAL* | *1* | *36* | *36* | *25.18* | *0.001* | *1* |  |  |  |
| 894 | MNG | Pinewood patches | 0.298 | 2 | 5.660 | 1.16 | 0.282 | 0.105 | 0.017 | 0.326 | None |
|  |  | Human facilities | 0.004 | 0 | 0.090 | 0 | 1 | 0 | 0 | 0.198 | None |
|  |  | Open shrublands | 0.333 | 7 | 6.320 | 0 | 1 | 0.368 | 0.190 | 0.591 | None |
|  |  | Orange tree crops | 0.241 | 9 | 4.580 | 1.34 | 0.247 | 0.474 | 0.273 | 0.683 | Selected |
|  |  | Other sites | 0.026 | 0 | 0.490 | 0 | 1 | 0 | 0 | 0.198 | None |
|  |  | Pasturelands | 0.088 | 0 | 1.670 | 0.28 | 0.596 | 0 | 0 | 0.198 | None |
|  |  | Roads | 0.008 | 1 | 0.160 | 0 | 1 | 0.053 | 0 | 0.265 | None |
|  |  | Sandy paths | 0.002 | 0 | 0.040 | 0 | 1 | 0 | 0 | 0.198 | None |
|  |  | *TOTAL* | *1* | *19* | *19* | *6.12* | *0.526* | *1* |  |  |  |
| 342 | NAT | Pinewood patches | 0.161 | 3 | 4.830 | 0.1 | 0.750 | 0.100 | 0.027 | 0.264 | None |
|  |  | Open shrublands | 0.532 | 9 | 15.950 | 2.43 | 0.119 | 0.300 | 0.165 | 0.480 | Avoided |
|  |  | Other sites | 0.166 | 3 | 4.970 | 0.14 | 0.712 | 0.100 | 0.027 | 0.264 | None |
|  |  | Pasturelands | 0.001 | 0 | 0.020 | 0 | 1 | 0 | 0 | 0.135 | None |
|  |  | Roads | 0.005 | 12 | 0.140 | 12.18 | 0.001 | 0.400 | 0.246 | 0.577 | Selected |
|  |  | Sandy paths | 0.026 | 2 | 0.780 | 0.02 | 0.893 | 0.067 | 0.008 | 0.224 | None |
|  |  | Lowland | 0.110 | 1 | 3.300 | 0.42 | 0.515 | 0.033 | 0 | 0.181 | None |
|  |  | *TOTAL* | *1* | *30* | *30* | *16.40* | *0.012* | *1* |  |  |  |
| 734 | NAT | Pinewood patches | 0.041 | 2 | 1.410 | 0 | 1 | 0.059 | 0.006 | 0.201 | None |
|  |  | Open shrublands | 0.330 | 6 | 11.210 | 1.38 | 0.240 | 0.176 | 0.080 | 0.339 | None |
|  |  | Other sites | 0.194 | 3 | 6.590 | 0.81 | 0.367 | 0.088 | 0.023 | 0.237 | None |
|  |  | Pasturelands | 0.068 | 2 | 2.300 | 0 | 1 | 0.059 | 0.006 | 0.201 | None |
|  |  | Roads | 0.003 | 5 | 0.090 | 3.25 | 0.072 | 0.147 | 0.060 | 0.306 | Selected |
|  |  | Sandy paths | 0.012 | 4 | 0.390 | 1.66 | 0.198 | 0.118 | 0.041 | 0.272 | Selected |
|  |  | Marshland | 0.277 | 11 | 9.400 | 0.03 | 0.874 | 0.324 | 0.190 | 0.493 | None |
|  |  | Lowland | 0.077 | 1 | 2.600 | 0.11 | 0.745 | 0.029 | 0 | 0.162 | None |
|  |  | *TOTAL* | *1* | *34* | *34* | *11.59* | *0.115* | *1* |  |  |  |
| 933 | NAT | Pinewood patches | 0.068 | 0 | 1.690 | 0.29 | 0.589 | 0 | 0 | 0.158 | None |
|  |  | Open shrublands | 0.503 | 7 | 12.580 | 1.76 | 0.185 | 0.280 | 0.141 | 0.478 | Avoided |
|  |  | Other sites | 0.293 | 2 | 7.320 | 2.46 | 0.117 | 0.080 | 0.011 | 0.261 | Avoided |
|  |  | Pasturelands | 0.002 | 0 | 0.060 | 0 | 1 | 0 | 0 | 0.158 | None |
|  |  | Roads | 0.010 | 16 | 0.260 | 19.80 | <0.001 | 0.640 | 0.444 | 0.798 | Selected |
|  |  | Sandy paths | 0.034 | 0 | 0.850 | 0 | 1 | 0 | 0 | 0.158 | None |
|  |  | Lowland | 0.090 | 0 | 2.240 | 0.72 | 0.397 | 0 | 0 | 0.158 | None |
|  |  | *TOTAL* | *1* | *25* | *25* | *24.70* | *<0.001* | *1* |  |  |  |
| 981 | NAT | Pinewood patches | 0.091 | 0 | 2.930 | 1.33 | 0.248 | 0 | 0 | 0.127 | None |
|  |  | Open shrublands | 0.719 | 22 | 23.000 | 0 | 1 | 0.688 | 0.513 | 0.822 | None |
|  |  | Other sites | 0.120 | 2 | 3.850 | 0.14 | 0.712 | 0.063 | 0.007 | 0.212 | None |
|  |  | Roads | 0.005 | 8 | 0.150 | 6.60 | 0.010 | 0.250 | 0.130 | 0.423 | Selected |
|  |  | Sandy paths | 0.026 | 0 | 0.840 | 0 | 1 | 0 | 0 | 0.127 | None |
|  |  | Lowland | 0.039 | 0 | 1.240 | 0.05 | 0.828 | 0 | 0 | 0.127 | None |
|  |  | *TOTAL* | *1* | *32* | *32* | *13.18* | *0.022* | *1* |  |  |  |
